# Target or barrier? The cell wall of early- and later-diverging plants *vs* cadmium toxicity: differences in the response mechanisms

**DOI:** 10.3389/fpls.2015.00133

**Published:** 2015-03-13

**Authors:** Luigi Parrotta, Gea Guerriero, Kjell Sergeant, Giampiero Cai, Jean-Francois Hausman

**Affiliations:** ^1^Dipartimento Scienze della Vita, Università di Siena, Siena, Italy; ^2^Environmental Research and Innovation, Luxembourg Institute of Science and Technology, Esch-sur-Alzette, Luxembourg

**Keywords:** cadmium, plant cell wall, heavy metal stress, heavy metal biosorption, cell wall polysaccharides, lignin

## Abstract

Increasing industrialization and urbanization result in emission of pollutants in the environment including toxic heavy metals, as cadmium and lead. Among the different heavy metals contaminating the environment, cadmium raises great concern, as it is ecotoxic and as such can heavily impact ecosystems. The cell wall is the first structure of plant cells to come in contact with heavy metals. Its composition, characterized by proteins, polysaccharides and in some instances lignin and other phenolic compounds, confers the ability to bind non-covalently and/or covalently heavy metals via functional groups. A strong body of evidence in the literature has shown the role of the cell wall in heavy metal response: it sequesters heavy metals, but at the same time its synthesis and composition can be severely affected. The present review analyzes the dual property of plant cell walls, i.e., barrier and target of heavy metals, by taking Cd toxicity as example. Following a summary of the known physiological and biochemical responses of plants to Cd, the review compares the wall-related mechanisms in early- and later-diverging land plants, by considering the diversity in cell wall composition. By doing so, common as well as unique response mechanisms to metal/cadmium toxicity are identified among plant phyla and discussed. After discussing the role of hyperaccumulators’ cell walls as a particular case, the review concludes by considering important aspects for plant engineering.

## INTRODUCTION

Plants, differently from animals, are sessile organisms and therefore cannot escape from potentially life-threatening conditions. This, in part, explains the great metabolic plasticity of plant cells, which have evolved mechanisms enabling them to adapt to and cope with environmental challenges. Plants growing on contaminated soils typically display either tolerance or avoidance. Plants tolerate heavy metals by sequestering them in specific plant organelles to keep them segregated from vital cellular components, or by the synthesis of enzymes involved in detoxification. Alternatively, the uptake and translocation of the heavy metal is decreased. However, there is a wide diversity in the details of the response, and species- or even clone-specific molecular responses have been recorded. Cadmium (Cd) is known to be phytotoxic and to affect plant physiological processes, from roots to shoots. Adverse effects caused by Cd are of considerable importance for all plants but, from an agricultural point of view, Cd is a toxic environmental pollutant that affects crop productivity (Du et al., 2012). While numerous reports have analyzed the cellular and enzymatic mechanisms involved in the response to Cd stress in plants, only a handful of studies have focused on the role of the plant cell wall. The cell wall is the outermost structure of plant cells and therefore the first in contact with the environment. It has important chemical characteristics, which make it a very good biosorbent of heavy metals. The presence of different functional groups (e.g., carboxyl, hydroxyl) deriving from the different wall polysaccharides favors for instance ion-exchange mechanisms with wall counter-ions. The cell wall is in its turn affected by heavy metals, since both its biosynthesis and composition can be altered. This leads to modifications in its physico-chemical properties which increase the binding capacity and at the same time lower the entry of heavy metals in the protoplast (Krzesłowska, 2011).

## IMPACT OF Cd POLLUTION ON PLANT CELLS AND ORGANS

Cd can have many consequences on plant physiology. It can affect both shoot growth and leaf biomass in *Zea mays* by impairing chlorophyll synthesis and by promoting the expression of defense proteins (Lagriffoul et al., 1998). In garlic (*Allium sativum*), Cd reduces root growth in a concentration-dependent manner (Liu et al., 2003).

It is also capable of damaging processes related to plant reproduction, with logical impacts on plant dispersal and biodiversity. For example, Cd can affect pollen germination and pollen tube growth by altering the polarization mechanism and by inducing abnormalities within the cell, such as blocking cytoplasmic streaming and alteration of the cytoplasmic organization (Sawidis, 2008). Moreover, in a recent study, the influence of exposure to a variety of heavy metals on the germination of 23 flax cultivar seeds was assessed. Using root elongation as the main parameter, large cultivar-dependent differences were found for some heavy metals (notably Cd, Ni and Co), while for other metals a more homogenous influence is described (Soudek et al., 2010).

The impact of Cd on crops can also be exerted by negatively influencing the translocation of nutrients, especially of minerals; in Cd-exposed tomato K, Fe, Mn, and Zn are reported to be poorly translocated in roots, while P and Mn uptake is drastically reduced in fruits (Moral et al., 1994). In pea, Cd has effects on both roots and leaves, and a significant inhibition of growth is combined with a reduction of transpiration and photosynthesis rate, as well as a general deterioration of the nutrient status (Sandalio et al., 2001). These are just a few examples describing the broad range of Cd-caused impacts on plants. For more information on the overall effects caused by Cd, readers can refer to specific reviews (Benavides et al., 2005; Qadir et al., 2014).

Cd is essentially absorbed by roots, but it localizes to all plant organs and tissues. Leaves are usually the preferential accumulation site of Cd. In leaves, Cd accumulates in different regions, which correspond to less metabolically active areas and to forthcoming necrotic regions. This suggests that an active metabolism might be required to detoxify Cd in plants and that the absence of detoxification mechanisms precedes necrotic events (Cosio et al., 2005). In the leaves of sensitive plants, Cd exerts its toxic activity by inducing chlorosis, thereby inhibiting growth. The effects caused by Cd range from morphological changes to reduction of the photosynthesis rate to decreased transpiration up to cell apoptosis (Souza et al., 2011).

Cd also affects the structure and function of roots. In *Allium cepa*, Cd treatment induces abnormalities, such as extensive vacuolization, condensation of the cytoplasm, damages to mitochondrial cristae, plasmolysis, and condensation of chromatin. Although dense granules were detected between the cell wall and the plasma membrane, no specific sites of Cd-accumulation were found in the cell walls of *Allium* roots (Liu and Kottke, 2004).

As plants are exposed to different environmental stresses, the effects of Cd contamination cannot be isolated from injuries caused by other environmental stresses and it might sometimes be difficult to predict the combinatory effect (Mittler, 2006). The simultaneous presence of different stresses might multiply the negative effects; however, as already observed in rice seedlings, one type of stress can also partially suppress the effects of another. In rice, for example, heat stress can ameliorate the negative effects of Cd pollution through the activation of protective systems based on anti-oxidant activities (Shah et al., 2013). However, the study of combined stresses is much less advanced than that of single stresses, although some studies have been performed combining Cd-exposure with other treatments (Sergeant et al., 2014). Given that polluted soils are generally not contaminated with Cd alone, the exposure of plants to mixtures of metals (Printz et al., 2013a,b), or the use of real-life polluted soils in experiments (Evlard et al., 2014a), could give new insights into the metabolic adjustments of plants when exposed to high concentrations of trace nutrients.

## ROLE OF PHYTOCHELATINS AND METALLOTHIONEINS IN THE RESPONSE OF PLANTS TO Cd STRESS

Although Cd is a generally toxic contaminant of the ecosystem, plants have evolved diverse mechanisms to respond to Cd contamination of their growth substrate. As for many compounds with detrimental effects on cellular metabolism, the toxicity of Cd is often alleviated through its sequestration to specific cellular compartments, such as vacuoles, or within specialized cells, such as trichomes (Harada et al., 2010). Adaptation of plants to Cd-contaminated soils might also rely on the symbiotic cooperation with other organisms. In *Medicago sativa*, colonization of roots by arbuscular mycorrhizae increases the tolerance of plants to Cd and the decreased Cd toxicity observed is somewhat proportional to the extent of colonization. It is likely that fungi possess a battery of enzymatic activities/mechanisms capable of chemically modifying Cd, thereby making it less toxic (Wang et al., 2012).

The cell wall can act effectively as a biosorbent of Cd, alleviating the toxic effects of this heavy metal. It is clear that the cell wall-based protection mechanism is only one of the processes used by plants to cope with the damage induced by Cd. Before exploring more specifically the molecular mechanisms in which the cell wall is involved, it is necessary to introduce the other protective mechanisms with which plants are equipped. These are based on specialized oligopeptides (namely phytochelatins and metallothioneins), on biochemical responses and on the intracellular sequestration of Cd.

Plants can respond by producing specific chelating agents, such as phytochelatins, that complex with Cd, thereby reducing its toxic potential (Carrier et al., 2003). The production of phytochelatins coincides with the activation of sulfur metabolism. This increases the synthesis of cysteine and of reduced glutathione (GSH), an antioxidant precursor of phytochelatins (Zhang and Shu, 2006). Increased levels of sulfur can also affect the distribution of Cd in plants by remobilizing it from the cell wall to the cytosolic compartment. As sulfur is important in the biosynthesis of sulfhydryl proteins, it is suggested that a higher content of sulfur generates larger amounts of proteins capable of sequestering Cd in the cytosol, thereby reducing the accumulation of the metal in the cell wall (Zhang et al., 2014c). Therefore, the availability of sulfur might control the synthesis rate of these sulfur-rich proteins (Loeffler et al., 1989; Uegsegger et al., 1990). Although this information is inferred from indirect observations (i.e., by determining that, in the presence of higher levels of sulfur, Cd is preferentially located in the vacuole), this also suggests that the balance between cell wall-bound and cytosol-associated Cd depends on the availability of proteins capable of sequestering Cd.

The use of phytochelatins for the detoxification of Cd-stressed plants is considered an important mechanism, but its actual role is still unclear and seems to be organ-dependent. Roots of the hyperaccumulator *Sedum alfredii* do not make efficient use of the phytochelatin-based mechanism for protection against Cd, while this process is conversely used in the shoots of the same plant (Zhang et al., 2010b). The use of different tolerance mechanisms in shoots and roots is also found in other hyperaccumulators such as *Typha angustifolia*, one based on GSH-related antioxidant systems (in leaves) and the other based on GSH-related chelation system (in roots; Xu et al., 2011).

An important class of cysteine-rich proteins binding heavy metals in plants and animals are metallothioneins (MTs). Their role in heavy metal stress in plants is known and their potential use in biotechnology is supported by recent studies, which have analyzed the effects of MTs overexpression in *Arabidopsis thaliana* and tobacco (Gu et al., 2014; Zhou et al., 2014). The transformed plants showed increased tolerance to Cd stress, which was accompanied by a lower accumulation of H_2_O_2_ in *A. thaliana* and by a higher ROS scavenging activity in tobacco.

Data in the literature indicate that a universal mechanism of Cd tolerance is not present and that different, often closely-related plants, respond differently when exposed to Cd. For instance, Cd-tolerant cultivars of black oat (*Avena strigosa*) accumulate Cd in the leaves, mainly in the cell wall. Those plants use a phytochelatin-based response when exposed to Cd, but also induce the up-regulation of ascorbate peroxidase and superoxide dismutase, indicating that an anti-oxidant response is triggered upon Cd treatment. By contrast, the phytochelatin-based response appears unimportant in Cd- sensitive plants, although their total content increased upon heavy metal exposure (Uraguchi et al., 2009).

## OTHER FACTORS AFFECTING THE RESPONSE/ADAPTATION/DEFENSE OF PLANTS TO Cd

The number and diversity of resistance mechanisms used by plants might be larger than expected. For example, it was reported that treatment of Cd-stressed plants with salicylic acid alleviates a number of effects caused by Cd. It is likely that the positive effects of salicylic acid are not directly correlated with the removal of Cd, but with the activation of response and defense genes, which in turn may code for enzymes involved in the maintenance of homeostasis of plant cells (Moussa and El-Gamal, 2010). Activation of pathogenesis-related defense proteins of the glucanase family is likely to be a common trait of many plants in response to Cd. This process was observed in different plants, for instance maize and soybean treated with Cd and other metals, suggesting that this defense mechanism is shared by relatively distant species (Piršelová et al., 2011).

The response of plants to Cd also requires the activation of enzymes capable of refolding proteins, as one of the most dramatic effects of intracellular Cd is protein denaturation. Therefore, some of the enzymes activated during response and adaptation to Cd belong to the chaperone family and chaperone-like proteins are likely active during the response to Cd pollution. As Cd can denature proteins, heat-shock proteins (HSPs) are involved in the refolding of denatured proteins and indeed levels of HSPs have been reported to increase after Cd treatment (Sergio et al., 2007). Increased levels of HSP70 were also detected in the bryophyte *Conocephalum conicum* exposed to Cd and Pb, suggesting that the need to refold proteins is a prerequisite to maintain cellular activity during heavy metal stress (Basile et al., 2013).

In pea roots, Cd and Cu treatment were reported to affect the enzymatic activity of proteins involved in the oxidation and peroxidation of cell wall components, such as guaiacol peroxidase, ascorbate peroxidase, coniferyl alcohol peroxidase, NADH oxidase and indole-3-acetic acid (IAA) oxidase (Chaoui et al., 2004). The evidence that Cd might increase the activity of cell wall-bound coniferyl alcohol peroxidase is of particular interest because this enzymatic activity metabolizes coniferyl alcohol, a monolignol used as substrate presumably involved in the lignification process (Quiroga et al., 2001). This suggests that Cd pollution might have severe repercussions on the development of secondary cell walls, by reducing their rigidity and robustness. An increase in guaiacol peroxidase activity was also reported in lichens exposed to higher concentrations of Cd (Sanità Di Toppi et al., 2005). As lichens are fungi-algae symbionts generally resistant and tolerant to heavy metal pollution, the use of plasma membrane-associated peroxidases might be a general mechanism to counteract the negative effects caused by Cd.

Changes in cell wall-associated peroxidase activity were also observed in roots of *Brassica juncea* stressed by Cd treatment. Here, Cd increases the activity of ionically cell wall-bound proteins, thereby increasing peroxidase activity too (Verma et al., 2008). The upregulated peroxidase activity is supposedly involved in the adaptation mechanism to Cd stress.

Although specific studies have not been conducted to determine the impact of Cd exposure on the cell wall proteome and on the diversity of responses in closely related species, numerous cell wall localized proteins were identified in general proteome studies on the impact of heavy metal pollution, namely Cd excess, on plants. These include general stress responsive proteins. For instance, in a study on two flax cultivars with different tolerance levels to Cd exposure, the cell wall-localized chitinase increased in both cultivars, but the protein accumulated at significantly higher levels in the more tolerant cultivar (Hradilova et al., 2010).

Proteomics studies in poplar found that exposure to Cd resulted in an increased accumulation of β-1,3-glucanase and chitinase (Kieffer et al., 2008; Durand et al., 2010). Moreover Kieffer et al. (2008) identified the significantly higher accumulation of different isoforms of cell wall localized peroxidase, linking the cell wall and anti-oxidative enzymes found in this compartment with Cd-stress. An intra-specific comparison of different *Populus nigra* ecotypes found differences in the mechanism of tolerance, indicating the variability and highlighting the difficulty to generate a physiological model, even at the species level. In Cd-stressed poplar plants, the activity of superoxide dismutase decreases, while the enzymatic activity of both peroxidase and catalase increased significantly in roots; this suggests that these plants trigger a response based on enhanced oxidase activity. Although the cytology of root cells is significantly altered, these plants exhibit a considerable capacity as hyperaccumulators and they have been consequently proposed as phytoremediators (Ge et al., 2012).

In addition to the various mechanisms outlined above, extracellular compartmentalization is another strategy used by many plants to limit the injuries caused by Cd. This mechanism involves the binding and accumulation of Cd in the cell wall, which serves as a reservoir for Cd. It is capable of concentrating and accumulating Cd, thus preventing it from penetrating inside the cell where it can cause damages. In the legume white lupin, the ability of the cell wall and vacuole-deposited phytochelatins to sequester Cd is relatively comparable in stems, but the cell wall is more effective in both roots and leaves (Vázquez et al., 2006). This suggests that these organs have specialized cell walls capable of sequestering Cd, while stems have mostly developed the phytochelatin-based mechanism for protection against Cd. Predominant accumulation of Cd in the cell wall of roots was also observed in *Spartina alterniflora* (Pan et al., 2012) and *Kandelia obovata* (Weng et al., 2012) treated with different concentrations of Cd. Enhanced accumulation of Cd in the cell wall of leaf cells was conversely reported in *Alternanthera philoxeroides* (Xu et al., 2012a).

Before discussing how Cd affects plant cell wall and how it can be a barrier against Cd, we need to introduce its key features.

## THE PLANT CELL WALL: A LIVING STRUCTURE

Plant cells are enveloped by a layer composed of structural proteins and polysaccharides (which can be impregnated by the aromatic polymer lignin), interwoven to form an intricate mesh, the cell wall. This structure has a complex three-dimensional organization and is considered an example of a natural biocomposite. The plant cell wall constitutes a material with exceptional mechanical performance and is taken as a model for the creation of materials with superior properties.

Cell walls protect the living protoplasts against external insults but, at the same time, they are very plastic since modifications in their composition and structure are responsible for enabling cell growth and development. Two types of cell walls can be distinguished: primary and secondary cell walls. The first is typically found in actively growing plant cells and is characterized by the presence of cellulose, pectin and hemicellulose. It is quite thin and flexible, thus enabling cell expansion (Guerriero et al., 2014a). Secondary walls are specialized structures synthesized when plant cells have ceased to elongate. They are responsible for mechanical strengthening (namely via the deposition of lignin) and accompany the phase of secondary growth, which determines the increase in stem girth (Guerriero et al., 2014a,b). The progressive stem thickening ensures resistance to bending. The main components of secondary walls are cellulose, xylan and lignin (Cosgrove and Jarvis, 2012).

A third type of wall (more specifically a wall layer) can be distinguished, which is found in specialized cell types and/or in response to mechanical stimuli: the gelatinous wall layer (G-layer). This type of wall is characterized by the presence of a thick layer of crystalline cellulose and is typically found in tension wood. Cells with a G-layer are also found in the stem of fiber crops like hemp, flax and nettle and are associated with the phloem. These cells are very long and known as bast fibers (Mellerowicz and Gorshkova, 2012; Guerriero et al., 2013).

Besides encasing and shielding plant protoplasts, the cell wall can be affected by nutritional stress and undergoes variations in composition. For example grapevine callus subjected to deficiency in S, N, and P showed walls with a decreased content in cellulose, increased lignin amount, alterations in the methylesterification of pectin (Fernandes et al., 2013). These results show how plant cell walls are remodeled in response to mineral deficits and how their composition is modified according to the stress condition applied. The plant cell wall indeed takes active part in transmitting exogenous signals to the interior of the cells: evidences show the existence of a cell wall integrity (CWI) maintenance mechanism which is triggered upon exogenous stresses (Hamann and Denness, 2011; Wolf et al., 2012; Engelsdorf and Hamann, 2014). The knowledge concerning CWI maintenance is far from being complete; however, given the similarity existing with that of yeast, both chemical and physical signals are supposed to contribute to it (Hamann and Denness, 2011). An example of a physical signal is represented by the weakening of the cell wall (for instance as a consequence to treatments with drugs like dichlobenil or isoxaben) up to a point that it can no longer counteract the internal turgor pressure with consequent cell swelling and bursting. A weakened wall causes stretching of the plasma membrane and activation of mechanosensitive channels which trigger an increase in the cytoplasmic concentration of calcium (Humphrey et al., 2007; Hamann and Denness, 2011).

Chemical signals are generated upon degradation of the cell wall by pathogens: for example, pectin-derived oligogalacturonides (OGAs) with a specific length, degree of acetylation and methylesterification, are capable of inducing rapid defense responses (Hamann and Denness, 2011; Vallarino and Osorio, 2012; Ferrari et al., 2013). The presence of signals, which allow the wall to “sense” the status of the cell, implies the existence of receptors that can intercept those signals. Several receptors have been described in the literature and among them wall associated kinases (WAKs) have been well studied. Those receptors are located in the plasma membrane, but have a domain protruding into the cell wall and a cytoplasmic kinase domain (Anderson et al., 2001; Humphrey et al., 2007). Their structure is therefore ideal to create a continuum cell wall-plasma membrane-cytoplasm and to transfer the signal from the outside to the inside of the cell. The role of these receptors is particularly interesting if one considers the relationship existing between heavy metal stress and water balance within plant cells. Gating of aquaporins was recorded within 10 min of heavy metal application to onion epidermal cells, which results in alterations in the turgor pressure (Przedpelska-Wasowicz and Wierzbicka, 2011). Since the WAKs membrane receptors are activated upon changes in turgor pressure (a phenomenon observed in pollen grains of *Picea wilsonii* after Cd stress; Wang et al., 2014), their functional study could provide important insights into the sensing mechanism and reveal downstream players involved in the signaling cascade.

## THE CELL WALL AS A BARRIER TO Cd

The composition of the cell wall shows many differences among phyla and it has played a vital role for their survival throughout evolution (Sarkar et al., 2009 and references therein). In the attempt to understand how different taxonomic groups respond to Cd (or heavy metals in general), it is valuable to compare the chemical composition and structure of cell walls from different phyla. Information might shed light either on alternative resistance mechanisms, or on how to improve the strategy used to tolerate Cd. The rationale at the basis of this assumption is that the variety of responses to Cd pollution, as found among different phyla, might be mirrored by molecular and cytological “tricks” that plants have evolved at the cell wall level. In discussing this issue, it is necessary to consider that evidence for the cell wall acting as a barrier against Cd (and other heavy metals) is not immediately clear from every study. The availability of molecular and structural data is low, which leads to fragmented pictures strongly affecting the development of a unifying model. Moreover, it is not possible to rule out that a unifying model does not exist and therefore it is necessary to present individual situations. The ability of either a plant cell or a plant *in toto* to tolerate Cd can be implemented at different levels and with different mechanisms. In the attempt to outline the different resistance mechanisms in which cell walls are involved, it is possible to rank them based on (a) the morphology of the barrier organ (for example the root), (b) the transport capacity of the contaminant, (c) the absorption of Cd into the cell wall by polysaccharides or proteins, (d) the impediment of Cd to penetrate through the plasma membrane.

The first part of the section aims at surveying the wall-related strategies developed by early- and later-diverging embryophytes. Charophytes, the closest algal relatives of embryophytes, are also briefly treated, given their importance in the study of land plant cell wall origin (Sørensen et al., 2011; Mikkelsen et al., 2014). The second part of the section discusses the mechanisms of Cd absorption/translocation in different plant organs and tissues, while considering the cell wall organization/composition.

### PLANT CELL WALL ACROSS DIFFERENT PHYLA: COMPOSITION AND MECHANISMS OF RESISTANCE TO Cd

Charophyte cell walls possess high amounts of mannose-containing hemicellulose, glucuronic acid, mannuronic acid, 3-*O*-methyl rhamnose (Popper and Fry, 2003) and possess neither lignin nor cutin. More importantly, they are capable of calcifying and calcite is known to bind and sequester heavy metals (Gomes and Asaeda, 2013). The macrophyte alga *Chara australis* can accumulate Cd and be used for remediation of contaminated soils (Clabeaux et al., 2013). Studies carried out on another species of macrophyte, *Chara fragilis*, showed that binding of uranyl species to the cell walls was mainly due to the presence of calcite (Daković et al., 2008). The biomineralization potential of Charophytes is therefore very promising for phycoremediation (i.e., the use of algae for the removal of pollutants) and further studies should be performed to link the cell wall composition of these algae to their calcifying potential.

Bryophytes, a group of early-diverging non-vascular plants, possess walls with mannose-containing hemicellulose, uronic acids and 3-*O*-methyl rhamnose, similarly to Charophytes (Sarkar et al., 2009). They lack lignin, although they contain lignans and other lignin-like polymers (Sarkar et al., 2009 and references therein). Bryophytes are known bioindicators of heavy metal pollution and their walls show high biosorption capacity because of the numerous ion-exchange sites (e.g., from uronic acids). The wall of the moss *Pohlia drummondii* was shown to form, together with the plasma membrane, an efficient shield against elevated zinc doses (Lang and Wernitznig, 2011); therefore, the living protoplast is protected from harmful effects. The moss *Scorpiurum circinatum* treated with different heavy metals, including Cd, immobilized the toxic ions in the cell walls, which is therefore used as the main detoxification site (Basile et al., 2012).

The Pteridophytes, another group of early-diverging plants, are very interesting organisms, as their cell walls show specialization to support the differentiation of a vascular tissue: galactomannan, glucomannan are abundant in secondary walls of leptosporangiates and lignin, together with xylan, has also been observed (Sarkar et al., 2009 and references therein). In the fern *Lygodium japonicum* it was shown that the wall pectins bind copper via homogalacturonans (Konno et al., 2005). In *Salvinia auriculata* Cd was shown to induce severe deformations at the cell wall level. Moreover an opaque layer could be observed along the middle lamella (Wolff et al., 2012). This finding might be explained by the binding capacity of pectins, the chief component of middle lamellas, a feature which is shared by seed plants. Among Pteridophytes, Equisetales certainly deserve attention, as their walls show the occurrence of mixed-linkage glucans and they are known for their biosilicification activity. Interestingly, a relationship was proposed between these hemicelluloses and Si mineralization (Fry et al., 2008). It would be interesting to investigate whether the sequestration of heavy metals by Equisetales takes place via mixed-linkage glucans.

These data indicate that the cell wall is a target for the accumulation of Cd in early-diverging plants, such as Bryophytes and Pteridophytes. These organisms use molecular mechanisms that are compatible with the chemical composition of their cell wall. However, as outlined above, both the structure and composition of the cell wall changed during evolution and it is therefore expected that chemically-different cell walls might respond differently to Cd pollution. The in-depth study of early-diverging plant cell wall biosynthesis and composition can reveal fine molecular details and inspire further avenues for improving the cell wall capacity to accumulate toxic heavy metals (see section “Future outlook”).

In later-diverging plants (such as spermatophytes), the increasing number of tissues and organs, coupled with the more sophisticated architecture, also generated cell walls with an increasing variability at the chemical and physical level. Consequently, it is likely that the diversity in Cd tolerance extends to the organ, tissue and even to the cellular level (see next paragraph).

In seed plants, and more specifically in flowering plants, most of the information was obtained from a limited number of species and, sometimes, on crop plants.

The evidence that the cell wall can exert a protective role was supported by studying Cd toxicity in crop plants. Pea plants were shown to be more sensitive than maize plants to Cd exposure; compared to pea plants, maize plants exhibited a higher percentage of cell wall-associated Cd, while pea showed higher content of Cd in the cytoplasm (Lozano-Rodríguez et al., 1997). This finding suggests that the cell wall acts as a barrier, preventing Cd from entering the cytoplasm where it is extremely toxic for plant cells. It is consequential that different cell wall structures and textures might bind Cd differently and thus provide different levels of protection. Comparable results were found in strawberry, where the treatment with different concentrations of Cd highlighted that the cell wall of leaves and roots is the primary reservoir for Cd, with root cell walls showing higher binding capacity than leaf cell walls (Zhang et al., 2010a). The fact that roots display a higher accumulating capacity than leaves can be an index of their protective activity preventing the entry and accumulation of Cd in the cytoplasm. If the concept of the cell wall as a barrier is true, the differential accumulating capacity of plant cell walls has to be found in their chemical and physical diversity. This diversity can cover not only the polysaccharide component of the cell wall, but also the protein fraction or modifications induced on one, the other or both components.

### PLANT CELL WALL COMPOSITION IN DIFFERENT ORGANS AND TISSUES: LINK WITH Cd ABSORPTION AND TRANSLOCATION

Since Cd is primarily adsorbed at the root level, it follows that the anatomy and molecular structure of the cell wall in root cells is a critical parameter. Findings in different plant species indicate that roots with higher content of suberin and lignin may be more impermeable to Cd and therefore more resistant to its absorption and translocation (Lux, 2010). Increased accumulation of suberin was observed in the roots of the monocotyledonous medicinal plant *Merwilla plumbea* exposed to Cd, a process interpreted as a protective response against penetration of Cd in the cells (Lux et al., 2011). The presence of root mucilage is another factor playing an important role in Cd adsorption, as shown in sunflower (Yang and Pan, 2013). The differential structure of roots might explain why Cd translocation is different between plants and variable even within the same taxonomic group.

Screening of different clones of *Salix* grown on identical real-life soil revealed remarkable differences in the translocation of Cd and other metals (Evlard et al., 2014b). Similar differences were found in solanaceous plants (Yamaguchi et al., 2011), different poplar species (He et al., 2013) and between ecotypes of the hyperaccumulators *Thlaspi caerulescens* and *T. praecox* (Xing et al., 2008).

In plants restraining Cd in roots, Cd is sequestered in the endoderm and the cortical regions of roots. When this filter fails, Cd can penetrate the xylem vessels and translocate to the shoot. Therefore, the specific composition of cell walls in the endoderm and cortex is critical (Akhter et al., 2014). Ultimately, the cell wall of roots is considered as an effective barrier against penetration of Cd and a relationship can be established between higher accumulation of Cd in the roots *vs* lower accumulation in the leaves (Sun et al., 2013). This suggests that Cd-tolerance goes through the capacity of roots to limit the diffusion of Cd towards the entire plant.

The protective activity exerted by the cell wall may not only depend on the polysaccharide fraction, but also on other cell wall molecules that can bind Cd and other heavy metals. As earlier suggested by Sanità Di Toppi and Gabbrielli (1999), the response of later-diverging plants to Cd may involve different types of molecular mechanisms, from the phytochelatin-based sequestration, to cell wall immobilization and plasma membrane exclusion, to the use of stress proteins. In this context, the cell wall (by acting as a molecular barrier) may be part of both adaptation mechanisms to permanent pollution and response mechanisms to acute stress. However, additional factors might be required to achieve full functionality and protection against Cd pollution. In fact, in addition to the binding of Cd to polysaccharides and proteins, tolerance to Cd might also involve the sequestration of Cd complexes in the cell wall. In crop plants such as maize and wheat, addition of P concomitantly to Cd treatment increased the tolerance of plants to Cd (Zhimin et al., 1999). P may associate with Cd thereby generating insoluble complexes that remain within the cell wall. Combination of binding to P and polysaccharides is also possible.

Therefore, the addition of P to contaminated soils seems to modify their physiochemical status, making Cd less available for plants, thereby improving the tolerance of plants to Cd pollution. In addition, Cd accumulates more consistently in the cell wall of plants growing on P-supplemented soils, which therefore hinder its entry in the cytoplasm (Siebers et al., 2013).

If the differential capacity of plants to resist Cd is linked to the chemical variability of their cell walls, it is clear that such variability can be consistently increased by modifying the protein component of the cell wall. Specific cell wall proteins could provide an additional barrier by binding Cd and thus preventing it from penetrating into the cytoplasm. In barley treated with Ni and/or Cd, the apoplastic fraction of leaves showed increased amount of apoplastic proteins that accumulate concomitantly with the metal treatment (Blinda et al., 1997). Therefore, the response of plants to contaminating metals may also involve the specific *de novo* synthesis of proteins that potentially make plants more tolerant. Proteins not directly involved in Cd binding have also been reported to increase Cd tolerance in plants. As previously discussed, an example is represented by cysteine-rich proteins as identified in *Digitaria ciliaris* and *Oryza sativa*. The genes coding for these proteins were found to be upregulated following Cd treatment and transgenic plants overexpressing cysteine-rich proteins are more tolerant to Cd stress by preventing entry into the cytoplasm (Kuramata et al., 2009). This finding also highlights the possibility of manipulating plants in order to increase their mechanisms of tolerance to pollutant agents.

Accumulation of Cd in the cell wall (or more generally in the apoplast) is also dependent on the efficient transport of Cd through the plasma membrane so that cytoplasmic Cd can be transported outside and incorporated in the cell wall. While doing so, the content of cytoplasmic Cd decreases while Cd accumulates in the cell wall. Transport of Cd through the plasma membrane is likely dependent on membrane transporters. Their involvement is also suggested by indirect evidence showing that, while Cd is promptly adsorbed in the cell wall, its influx in the symplast is linear with Cd concentration, which suggests the activity of low-affinity transport systems (Redjala et al., 2009). Recently the transporter IRT1 (IRON-REGULATED TRANSPORTER 1) was shown to be not only involved in the uptake of iron, but also of toxic metals, among which Cd (Barberon et al., 2014). This root transporter is recycled via a mechanism involving a phosphatidylinositol-3-phosphate-binding protein, FYVE1, which controls its delivery to the outer plasma membrane domain of root epidermal cells.

These observations stress the importance of studying the cell wall structure of both tolerant and resistant plants in order to identify those molecules that provide the highest protection against Cd. One example of an important molecule is lignin. The protective activity of lignin is suggested by pretreatment of wheat plants with wheat germ agglutinin, which generally exerts an amelioration of Cd effects. This pretreatment likely involves a different balance between hormone levels but an important phenotypic effect is an accelerated lignification of cell walls (Bezrukova et al., 2011). Such secondary modifications might render cell walls less permeable to Cd thus limiting its entry in the cell. Lignin can also bind Cd thereby inhibiting its diffusion into the cells. Higher abundance of lignin in secondary cell walls might thus make plants more tolerant to the effects of Cd treatment.

**FIGURE 1:**
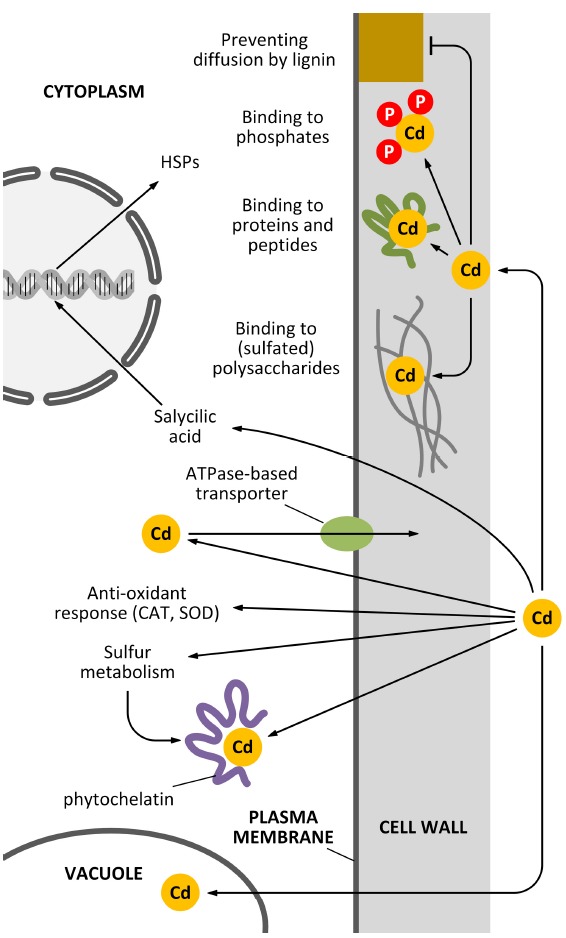
**How the cell wall can act as a barrier against Cd pollution.** Plant cells can counteract the toxicity of Cd in several ways and the cell wall actively participates to most of these mechanisms. Apart from mechanisms of sequestration based on the activity of vacuoles and phytochelatins, additional decontaminating activities can be exerted by the cell wall. For example, specific polysaccharides of the cell wall can bind Cd through sulfated moieties (although this situation is restricted to very few cases, e.g., seaweeds and vascular plants adapted to inhabit saline environments). Cd might also be sequestered in the cell wall by the activity of either cell wall-associated proteins or cell wall phosphates. An additional barrier might be represented by secondary modifications of the cell wall, such as lignin deposition.

In *Pisum sativum* leaves, lignin was shown to be deposited following Cd stress and this process was accompanied by an oxidative burst in the xylem vessel cell walls (Rodríguez-Serrano et al., 2009). Moreover, Cd was shown to favor the process of xylogenesis, by inducing the activity of enzymes via H_2_O_2_ accumulation (Schützendübel et al., 2001). The use of sophisticated labeling techniques involving quantum dot technologies can highlight the interaction between Cd and specific cell wall components (Djikanovic et al., 2012). The multitude of activities of the cell wall as a barrier against Cd pollution is schematically summarized in Figure 1.

## THE CELL WALL AS TARGET: EFFECTS OF Cd ON CELL WALL SYNTHESIS AND COMPOSITION

In the previous paragraphs, the cell wall has been considered as a potential reservoir for Cd and thus as an efficient barrier to its penetration in the plant. In some cases, Cd is not efficiently neutralized and it might become an injuring factor by affecting the structure of cell walls. The toxic activity of Cd can be exerted at different levels. Cd can alter the physical structure of the cell wall by interacting with negatively charged molecules (i.e., carboxyl groups and sulfates). In this way, Cd can substantially modify the cell wall resistance to turgor pressure thereby making the cells weaker. In addition to interacting with the polysaccharide component, Cd can interfere with the enzymatic activities present in the cell walls by inhibiting the enzymatic reactions that strengthen the cell wall structure. Furthermore, Cd can affect directly or indirectly the synthesis of cell wall components, or interfere with the transport of the latter to the final destination.

Presence of Cd also affects the deposition pattern of pectins. In the hypocotyl of *Linum usitatissimum*, the presence of an excess of Cd changes the ratio between low and high methyl-esterified pectins, causing the former to accumulate consistently in epidermal cells and determining the collapse of the sub-epidermal cell layer (Douchiche et al., 2007). This unbalance affects the primary walls and may cause swelling of hypocotyl tissues. It is not clear how Cd determines such effects, but it is likely that it alters the expression level of pectin-metabolizing enzymes such as pectin methyl-esterase (PME). Overexpression of this enzyme can change the ratio between low and high methyl-esterified pectins by altering their level of esterification. This leads to a relatively higher abundance of acidic pectins and extremely impairs the shaping of plant cells, which is based on a precise balance in degree of esterification. In addition to changing the degree of esterification of pectins, Cd can also affect more generally the chemistry of these polysaccharides for example by altering the expression of peroxidases, which in their turn alter the chemical structure of homogalacturonans (Paynel et al., 2009). The effects caused by Cd on the development of cell walls and, more specifically, on pectins might be more subtle and not immediately appreciable. This is the case for flax fibers where Cd treatment affects the deposition of secondary cell walls in terms of adhesion of cellulose microfibrils. On the contrary, the effects on both low and high methyl-esterified pectins are not immediately explicable (Douchiche et al., 2011).

That pectins are important in binding Cd is demonstrated by experiments with plants grown in P deficiency. Lower levels of P alleviate the negative effects caused by Cd because P reduces the content of pectins and PME in the cell wall. The reduced level of this enzyme enhances the ratio between low and high methyl-esterified pectins further, making a lower number of active sites available for Cd binding, thereby triggering a heavy metal-exclusion mechanism (Zhu et al., 2012).

Further studies on the role of pectins in response to Cd exposure were carried out in rice. The addition of NO donors concurrently with Cd treatment makes plants more resistant to Cd injuries. The resistance is mainly based on the overproduction of pectins and on the simultaneous reduction of cellulose levels (Xiong et al., 2009). It is likely that pectins are Cd adsorbent via carboxylic groups, thus preventing the metal from entering the cytoplasm. It would be fascinating to analyze if NO donors alter the expression of genes coding for enzymes involved in pectin synthesis. The possible role of NO in alleviating the symptoms caused by Cd treatment has been already reviewed (Xiong et al., 2010).

The evidence that toxic elements such as heavy metals could bind to pectin in the cell walls has been already observed and described (Malovikova and Kohn, 1982). There is a vast literature on the chemical interaction between heavy metals and pectins (Krzesłowska, 2011) although the exact mechanism of damage caused by Cd on pectins is not completely clear. It is likely that Cd can substitute calcium ions thereby modifying the rigidity of the pectin skeleton. This fact has been suggested in the alga *Ulva lactuca* where the rhamnose subunits are cross-linked by calcium and the replacement of calcium by Cd can decrease the rigidity of the cell wall (Webster and Gadd, 1996). It is noteworthy that calcium can alleviate the injuries caused by Cd, likely either because calcium can compete for the binding to cell wall polysaccharides, or Cd might use calcium channels to penetrate inside cells (Suzuki, 2005). At the cellular level, Cd can induce remarkable aberrations on the cell wall structure resulting in changes in cell shape (Sawidis and Reiss, 1995; Sawidis, 2008). These observations are consistent with the hypothesis that Cd interferes with the process of cell wall structuring probably by altering the exact arrangement of pectins. In some experimental cases, exposure to soils contaminated with Cd induces a general increase of the levels of pectin and a reduction of methyl-esterified pectins (Astier et al., 2014). Nevertheless, understanding exactly the alterations induced by Cd on the structure of the pectin skeleton is not simple and the use of antibodies directed against different chemical forms of pectin cannot help to define exactly how Cd damages the pectin cell wall (Douchiche et al., 2011).

Direct effects of Cd on the synthesis of other polysaccharides (such as cellulose and callose) are not known. However, it was reported that Cd triggers the accumulation of specific Cd-induced glycine-rich proteins (cdiGRPs). These are cell wall-associated proteins that might positively regulate the synthesis of callose. The cdiGRPs likely work in cooperation with an additional cell wall protein, the so-called GrIP (cdiGRP-interacting protein; Ueki and Citovsky, 2005). The model proposed by the authors suggests that Cd increases the post-translational accumulation of cdiGRP, which is also dependent on the expression of GrIP. Once accumulated in the cell wall, cdiGRP enhances the production and/or accumulation of callose, which consequently represents a cell wall adaptation to Cd pollution. Accumulation of callose in response to metal stress probably does not involve altered mechanisms of callose removal. In maize and soybean, treatment with metals (including Cd) induces the accumulation of callose in the cell wall. Results indicate that the accumulation is not related to decreased enzymatic activity of glucanases and reinforce the hypothesis that accumulation of callose is primarily dependent on a higher rate of synthesis and/or deposition (Piršelová et al., 2012). Like callose, there are few reports on the relationship between Cd and cellulose and the relationship between Cd pollution and cellulose synthesis is far from being completely understood.

The effects of Cd on cell wall synthesis might also be indirect. Cd is known to affect the level of apoplastic sucrose probably by altering the activity of cell wall invertase. Consequently, sucrose accumulates in the cell wall and decreases in the cytoplasm (Podazza et al., 2006). Lower levels of cytoplasmic sucrose can have detrimental effects on cell wall synthesis by making substrates for the biosynthesis of cell wall precursors less available. Synthesis of both cellulose and callose requires the addition of UDP-glucose, which can be produced by two different metabolic pathways. The first requires the enzyme UDP-glucose pyrophosphorylase (UGPase) that catalyzes the reversible production of UDP-glucose and pyrophosphate from glucose-1-phosphate and UTP; the second mechanism involves the activity of sucrose synthase, which breaks down sucrose (in the presence of UTP) into UDP-glucose and fructose (Kleczkowski et al., 2010). Lower levels of cytoplasmic sucrose may reduce the activity of sucrose synthase and consequently may lower the production rate of UDP-glucose. As sucrose synthase is associated with both callose synthase and cellulose synthase (Brill et al., 2011), it follows that the Cd-induced reduction of cytoplasmic sucrose might affect the synthesis of cellulose and callose.

**FIGURE 2:**
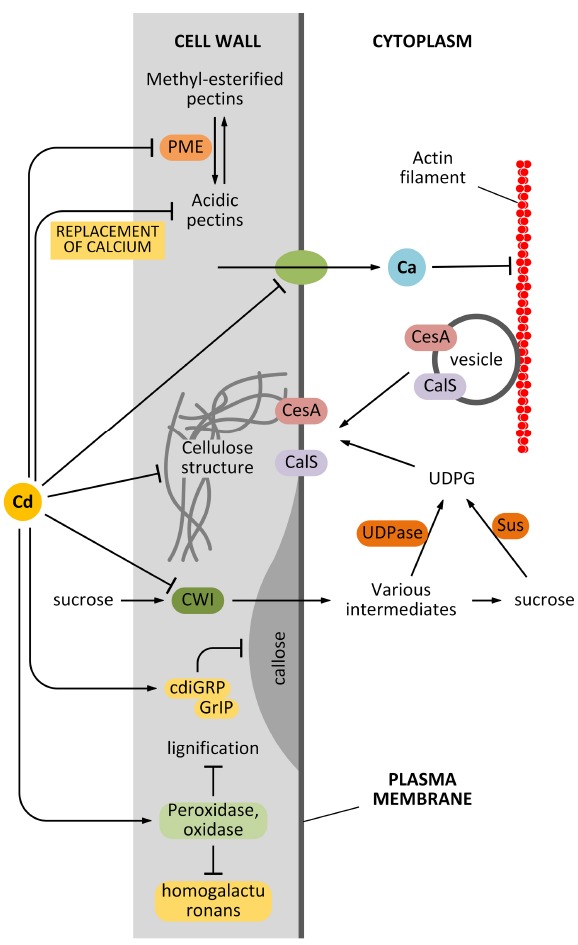
**Effects of Cd on cell wall synthesis.** Cd might affect the synthesis of cell wall components in many different ways. First, Cd can affect the activity of pectin methyl-esterase (PME) that consequently affects the balance between methyl-esterified and unesterified pectins. In turn, this alters the elasticity of the cell wall. Second, Cd might affect the structure of cellulose directly or indirectly by affecting the delivery of cellulose synthase (CesA). The indirect interfering activity can be exerted by damaging the actin filament structure or by affecting the uptake of sucrose (which provides the metabolites for cell wall synthesis). Cd is also hypothesized to affect the synthesis of callose by interfering with regulatory proteins. Finally, Cd can also modify the lignification level of the cell wall, by acting on related enzymes.

Another pathway by which Cd can affect the synthesis and deposition of cell wall is indirect and relates to the effects of Cd on cytoplasmic streaming. It is known that delivery of cell wall synthesizing enzymes requires an intact cytoskeleton and an intact membrane system: cell wall synthesizing enzymes traffic along the cytoskeleton, which guides the enzymes towards the final insertion sites in the plasma membrane (Crowell et al., 2010). The precise balance between insertion and removal of cell wall synthesizing enzymes is critical to correctly deposit the cell wall and to assemble a proper cell wall texture. Cd affects the intracytoplasmic movement of organelles and vesicles in the epidermal cells of the model plant *A. cepa* (Wierzbicka et al., 2007). Cytoplasmic streaming disorders are likely to impact negatively on the cell wall assembly. We do not know how Cd affects organelle streaming, if through an unspecific pathway (for example, by altering the ionic homeostasis of cells), or a more specific mechanism that directly inhibits the proteins involved in organelle transport. Nevertheless, it is clear that Cd has a considerable influence on the structure of actin filaments and consequently on cytoplasmic streaming. In *A. thaliana* root hairs, Cd alters the balance between influx and efflux of Ca^2+^, which consequently abolishes the Ca^2+^ gradient. Since Ca^2+^ regulates the dynamics of actin filaments in tip-growing cells, the Cd-induced imbalance of Ca^2+^ has negative effects on actin filaments that consecutively impair the motility of organelles and vesicles, as reviewed by Wan and Zhang (2012). As observed by the authors, the cell wall structure is also affected because of the altered cytoplasmic streaming (Fan et al., 2011). The relationship between Cd and Ca^2+^ is likely more complex than expected in view of the finding that a member of the plant cadmium-resistance (PCR) protein family is a Ca^2+^ efflux transporter, which is involved in the finer regulation of intracellular Ca^2+^ concentration (Song et al., 2011). The global effects of Cd on cell wall synthesis are summarized in Figure 2.

## Cd ACCUMULATION IN PLANTS: THE CASE OF HYPERACCUMULATORS AND THE EFFECT OF NUTRIENTS

In the previous sections, data concerning how Cd can be a harmful element for plants and how plants can respond to damage caused by Cd were presented and discussed. Emphasis was put on how evolutionarily-distant plants can respond in different ways to Cd treatment; this is an indication that several different mechanisms have evolved in response to Cd. In this section, the focus will be put on those plants showing heavy metal hyperaccumulating capacity, since they represent an extreme case. Moreover, the study of their cell walls can lead to important observations, which can be transferred to biotechnological approaches. In this section considerations on the role of macro- and micro-nutrients in the increased Cd accumulation/tolerance will also be made, since this aspect is linked with the potential of enhancing the plant capacity to accumulate Cd.

Hyperaccumulator plants are characterized by the extraordinary capacity of accumulating Cd and of translocating it towards the aerial parts of plants (namely leaves) and of detoxifying the element locally (Rascio and Navari-Izzo, 2011). At the level of leaves, Cd can be accumulated in specific sites, such as the vacuoles or the cell wall. It is therefore possible to assume that in hyperaccumulators the ability of the cell wall to absorb Cd is so high that Cd fails to actively penetrate inside the cytoplasm and remains confined within the cell wall. Even if the molecular mechanisms working in hyperaccumulator plants are the same as those present in Cd-resistant plants, the main difference is that the former can concentrate Cd in the cell wall at extreme levels without apparent damage. There are many examples of hyperaccumulators and, as the study is continuing, new species are added to this list. The root hairs of *T. caerulescens*, a metal hyperaccumulator, can accumulate consistent levels of Cd in their cell walls thereby preventing Cd from penetrating in the cytoplasm (Nedelkoska and Doran, 2000). Massive accumulation of Cd in hyperaccumulator plants is also based on complexes with phosphates within the cell wall. However, not all cell types accumulate Cd at the same level (Küpper et al., 2000). Accumulation of Cd and Zn may have particular relevance in hyperaccumulator plants, such as *Sedum plumbizincicola*, in which heavy metals are chiefly accumulated in shoots and relatively less in roots (Cao et al., 2014). *Dittrichia viscosa* is another model-accumulator plant that accumulates large amounts of Cd in the cell wall (Fernández et al., 2014).

The ability of certain species to accumulate Cd is linked not only to the higher ROS scavenging activity, but also to Fe homeostasis: studies carried out on the Cd-accumulator *Solanum nigrum* and the low Cd-accumulating *Solanum torvum* showed that Cd treatment caused a lower Fe accumulation in *S. torvum* compared to *S. nigrum* (Xu et al., 2012b). This difference is related to the increased expression of Fe transporters, namely IRT1, and IRT2, in the roots of the Cd-accumulator *S. nigrum* (Xu et al., 2012b).

The most important application of hyperaccumulator plants is linked to the possibility of decontaminating polluted sites (see also “Future outlook”). Consequently, researchers are looking for hyperaccumulators that can be used to detoxify contaminated soils in an economically feasible and reasonable way. This has been already proposed for two species of *Iris* (Han et al., 2007). Those plants, capable of restoring optimal conditions in contaminated soils, have evolved molecular mechanisms to tolerate high concentrations of contaminants. The acquisition of tolerance to contaminants involves the development of mechanisms for the accumulation of pollutants in specific cell sites, such as the vacuole and cell wall of root cells where Cd might be complexed with insoluble phosphates that strongly limit the translocation of Cd to aboveground tissues and organs (Zhang et al., 2014b). This suggests that the hyperaccumulating capacity of cell walls can be induced or improved by adding specific chemical groups. This can involve both the polysaccharide and the protein component of the cell wall. In this respect, studies on transgenic tobacco plants overexpressing a xyloglucan endotransglucosylase/hydrolase gene support this statement. In those plants, xyloglucans accumulated at lower levels, which prevents plants from binding Cd. This suggests that xyloglucans could be relevant binding sites for Cd and are involved in Cd tolerance; conversely, plants with lower levels of xyloglucans accumulate lower amounts of this heavy metal (Han et al., 2014).

Another significant example is represented by the addition of Si. Si considerably reduced the net influx of Cd by likely producing Si-cell wall complexes that efficiently adsorb Cd thus preventing its entry in the cytoplasm (Liu et al., 2013). In such a way, the cell wall can be potentiated, thus becoming a more effective barrier against Cd. The ameliorating activity of Si is dependent on its concentration, as well as on the concentration of Cd, and Si has a major effect on Cd influx by modifying the cell wall texture. However, Si does not affect intracellular processes like photosynthesis (Lukacová et al., 2013). In mangrove plants, Si is known to alleviate the effects of Cd pollution by restricting Cd to the cell wall of roots and reducing the concentration of Cd in the symplast. Therefore Si enhances the capacity of cell walls to restrain Cd, thereby limiting its diffusion (Zhang et al., 2014a).

The possibility of improving the hyperaccumulator capacity of plants may not be simple, as the number of genes involved may be huge. A preliminary assay in the hyperaccumulator *S. alfredii* revealed that plants under Cd stress respond by modifying the expression level of more than 100 genes responsible for different functions (including cell wall modification; Gao et al., 2013). Comparable analyses by using suppression subtractive hybridization screens showed that the activity of several genes is important in making plants of *Salix caprea* tolerant to Cd pollution. Some of the investigated genes belong to the family of cell wall modifying enzymes (Konlechner et al., 2013). This finding opens the way to the identification of potential genes (and gene products) capable of raising the tolerance capacity of plants against Cd.

The study of hyperaccumulators’ cell walls will lead to the identification of those functional chemical groups capable of absorbing high amounts of Cd thereby preventing its entry in the cytoplasm. In addition to understanding the chemistry of this process, it is also necessary to identify the cellular mechanisms allowing the addition of such chemical groups in the cell wall (e.g., specific enzymes that modify the chemical structure of cell wall components). It follows that the development of hyperaccumulator plants requires a high level of knowledge. Ideally, the most convenient result would be to combine the hyperaccumulating capacity of non-crop plants (in which this process has been widely studied) with the economically advantageous crop plants. By transferring the genetic information at the base of Cd resistance into crop plants, it will be possible to design improved plants capable at the same time to detoxify the soil and to maintain the soil productivity rate (Wu et al., 2010).

## PLANT BIOTECHNOLOGY AND Cd TOXICITY: WHAT SHOULD BE CONSIDERED?

The examples listed previously show that plants are equipped with different mechanisms to cope with the toxicity induced by heavy metals (Cd, specifically). Studying the variety of responses will allow us to identify specific mechanisms used to tolerate large doses of pollutant. Those mechanisms (based either on chelating proteins, on general biochemical responses, or on sequestering in the vacuole/cell wall) may not necessarily be present in plants of economic interest and may concern either plant products or plant waste. For example, an interesting applicative aspect concerning the detoxifying ability of cell walls is the use of waste vegetable components rich in pectins as biosorbent of heavy metals (Wan Ngah and Hanafiah, 2008). The knowledge of these biosorbent mechanisms can be used to develop plants capable of sequestering Cd (hyperaccumulators; Douchiche et al., 2010). Pectins can also find applications outside the plant context. Fruits rich in pectin can absorb significant amounts of heavy metals (Schiewer and Patil, 2008). Such technologies can be extended to pectin gels extracted from commercial plants, such as sugar beet. Such studies, in addition to demonstrating that pectin-enriched matrices can actually be applied for Cd removal, confirm that Cd binds pectin matrices by removing calcium ions (Mata et al., 2009, 2010). Other food wastes have been proposed in this regard, for example citrus peels (Schiewer and Iqbal, 2010).

Several studies have shown altered Cd accumulation in plants engineered to express genes involved in heavy metal uptake, translocation or chelation. For example, as previously mentioned, tobacco plants expressing the Zn and Cd translocator *HMA4* from *A. thaliana*, which controls translocation to the shoot (Wong and Cobbett, 2009), show decreased Cd accumulation, but also deep transcriptional remodeling leading to increased lignification (Siemianowski et al., 2014). Restricting the examples to the cell wall, which is the topic of this review, the upregulation of a peroxidase, *O*-methyltransferase and hydroxycinnamoyl reductase leads to an increased deposition of lignin in a specific layer between the root epidermis and the first cortical layer, which consequently blocks Cd apoplastic movement towards the stele (Siemianowski et al., 2014). These transgenic tobacco plants exposed to Cd, however, show an enhanced Fe and Zn deficiency status linked to the overexpression of two metal uptake genes, *ZIP1* and *IRT1* (Siemianowski et al., 2014). These results show how challenging biotechnological prospects for improving heavy metal tolerance in plants are, as plants overexpressing a specific gene might show unfavorable/unexpected features linked to the response of the host to the transgene.

The insertion of new genes whose products change the chemical structure of cell wall might be a way to engineer plants that are more Cd-resistant. An alternative strategy would be to introduce specific mutations in genes already present. Mutation in one gene coding for a specific cellulose synthase subunit significantly alters the content of cellulose and decreases the cell wall thickness in rice plants. Although there were no clear phenotypical differences in comparison to control plants, mutant plants showed a significant decrease in the translocation rate of Cd along xylem vessels, which appeared as abnormal in shape. The new morphological structure of xylem, although not impairing the total shape of plants, determines a consistent reduction in the transport of Cd to leaves, thereby making rice plants more tolerant to Cd pollution (Song et al., 2013). Together with other studies, this finding highlighted that the translocation of Cd from roots to shoots is likely more critical than the mere uptake of Cd at the root level (Xin et al., 2013). Therefore, the accumulation of Cd in the leaves seems more closely related to the translocation rate than to Cd uptake and reinforces the necessity of studying how Cd is translocated through the xylem and the factors that can be modified/regulated to delay such process.

The presence of the so-called “metal cross-homeostasis” has been identified as a primary factor influencing the phenotype of plants with a modified expression of genes involved in metal homeostasis (Antosiewicz et al., 2014). This cross-homeostasis is primarily due to the cross-talk existing between the homeostatic mechanisms of different metals: for example the concentration of different metals regulates the same players of the transcriptional wiring, the same transporters and chelators (Antosiewicz et al., 2014).

Approaches in biotechnology represent the most effective way to engineer heavy metal accumulation in plants. In particular, three elements are relevant to devise effective engineering approaches for heavy metal uptake/accumulation in plants: (1) the choice of specific promoters, for instance tissue-specific promoters (Antosiewicz et al., 2014), which restrict eventual modifications to a specific tissue or organ; (2) integrative studies aimed at deciphering the response of plants to a specific metal at a transcriptional, protein and metabolic level; (3) the choice of the host to express a specific transgene. This last feature is very relevant, as fast-growing plants with high biomass yield should be preferred. As an example, fiber crops like hemp (*Cannabis sativa* L.) represent very good candidates for engineering approaches. Hemp has a deep root system, a fast growth rate and is known to require less water than other crops (like cotton): all these features make it extremely interesting for biotechnology. Moreover the hemp genome has been sequenced (van Bakel et al., 2011), a feature which can greatly favor the design of constructs, e.g., for synthetic biology. Last but not least, the tissues of hemp stems show a great diversity of chemical composition: the core is lignified, while the cortex is rich in cellulosic bast fibers. This heterogeneity in cell wall composition offers a variety of functional groups potentially useful for heavy metal sequestration in hemp stem tissues.

## FUTURE OUTLOOK: WHAT COMES NEXT?

To conclude this review, a list of points that need further study is hereafter proposed to achieve a more comprehensive knowledge of the mechanisms involved in Cd toxicity in plants and to devise new strategies for the biotechnological improvement of plant tolerance to toxic heavy metals.

(1) Studying how early- and later-diverging plants progressively adapted and responded to Cd will reveal a particular mechanism or even a combination of molecular mechanisms, which could be used to improve the response of crop plants grown on contaminated soils. In this context the coupling of–*omics* sciences (transcriptomics, proteomics, metabolomics) and advanced imaging techniques (namely mass spectrometry imaging, like Secondary Ion Mass Spectrometry, SIMS) can generate a wealth of information covering different levels of biological complexity.
(2) Considering the chemical variety of cell walls and the potential diversity in polysaccharide and protein composition, there might be several possibilities for the cell wall to interfere with the toxic activity of Cd. Therefore, the study of cell walls (in terms of assembly and composition) in hyperaccumulators can provide important information on how to improve the survival or the productivity of plants growing on Cd-contaminated soils. In this context, it is important to identify the natural targets of Cd at the cell wall level and to figure out which of these targets can be modified to prevent the negative effects of Cd. One approach to develop this point is the functional study of those targets via generation of mutants/overexpressors. The purpose of this approach is to generate plants that either cannot absorb Cd or can sequester Cd in the cell wall thereby reducing or avoiding detrimental effects in terms of growth or productivity.
(3) Studies on the effects induced at the cell wall-level by the application of beneficial mineral nutrients deserve further attention. In particular the role of Si and P in alleviating the toxic effects of Cd should be analyzed via an integrative biology approach to identify the targets of this interaction.

### Conflict of Interest Statement

The authors declare that the research was conducted in the absence of any commercial or financial relationships that could be construed as a potential conflict of interest.

## References

[B1] Akhter M. F. Omelon C. R. Gordon R. A. Moser D. Macfie S. M. (2014). Localization and chemical speciation of cadmium in the roots of barley and lettuce. Environ. Exp. Bot. 100, 10–19 10.1016/j.envexpbot.2013.12.005

[B2] Anderson C. M. Wagner T. A. Perret M. He Z. H. He D. Kohorn B. D. (2001). WAKs: cell wall-associated kinases linking the cytoplasm to the extracellular matrix. Plant Mol. Biol. 47, 197–206. 10.1023/A:1010691701578 11554472

[B3] Antosiewicz D. M. Barabasz A. Siemianowski O. (2014). Phenotypic and molecular consequences of overexpression of metal-homeostasis genes. Front. Plant Sci. 5:80. 10.3389/fpls.2014.00080 24639682 PMC3945530

[B4] Astier C. Gloaguen V. Faugeron C. (2014). Phytoremediation of cadmium-contaminated soils by young douglas fir trees: effects of cadmium exposure on cell wall composition. Int. J. Phytorem. 16, 790–803. 10.1080/15226514.2013.856849 24933885

[B5] Barberon M. Dubeaux G. Kolb C. Isono E. Zelazny E. Vert G. (2014). Polarization of IRON-REGULATED TRANSPORTER 1 (IRT1) to the plant-soil interface plays crucial role in metal homeostasis. Proc. Natl. Acad. Sci. U.S.A. 111, 8293–8298. 10.1073/pnas.1402262111 24843126 PMC4050562

[B6] Basile A. Sorbo S. Conte B. Cardi M. Esposito S. (2013). Ultrastructural changes and Heat Shock Proteins 70 induced by atmospheric pollution are similar to the effects observed under in vitro heavy metals stress in *Conocephalum conicum* (Marchantiales–Bryophyta). Environ. Pollut. 182, 209–216. 10.1016/j.envpol.2013.07.014 23933125

[B7] Basile A. Sorbo S. Pisani T. Paoli L. Munzi S. Loppi S. (2012). Bioacumulation and ultrastructural effects of Cd, Cu, Pb, and Zn in the moss *Scorpiurum circinatum* (Brid.) Fleisch. Loeske. Environ. Pollut. 166, 208–211. 10.1016/j.envpol.2012.03.018 22516710

[B8] Benavides M. P. Gallego S. M. Tomaro M. L. (2005). Cadmium toxicity in plants. Braz. J. Plant Physiol. 17, 21–34 10.1590/S1677-04202005000100003

[B9] Bezrukova M. V. Fatkhutdinova R. A. Lubyanova A. R. Murzabaev A. R. Fedyaev V. V. Shakirova F. M. (2011). Lectin involvement in the development of wheat tolerance to cadmium toxicity. Russ. J. Plant Physiol. 58, 1048–1054 10.1134/S1021443711060021

[B10] Blinda A. Koch B. Ramanjulu S. Dietz K. J. (1997). De novo synthesis and accumulation of apoplastic proteins in leaves of heavy metal-exposed barley seedlings. Plant Cell Environ. 20, 969–981 10.1111/j.1365-3040.1997.tb00674.x

[B11] Brill E. van Thournout M. White R. G. Llewellyn D. Campbell P. M. Engelen S. (2011). A novel isoform of sucrose synthase is targeted to the cell wall during secondary cell wall synthesis in cotton fiber. Plant Physiol. 157, 40–54. 10.1104/pp.111.178574 21757635 PMC3165887

[B12] Cao D. Zhang H. Wang Y. Zheng L. (2014). Accumulation and distribution characteristics of zinc and cadmium in the hyperaccumulator plant *Sedum plumbizincicola*. Bull. Environ. Contam. Toxicol. 93, 171–176. 10.1007/s00128-014-1284-8 24789526

[B13] Carrier P. Baryla A. Havaux M. (2003). Cadmium distribution and microlocalization in oilseed rape (Brassica napus) after long-term growth on cadmium-contaminated soil. Planta 216, 939–950. 10.1007/s00425-002-0947-6 12687361

[B14] Chaoui A. Jarrar B. El Ferjani E. (2004). Effects of cadmium and copper on peroxidase, NADH oxidase and IAA oxidase activities in cell wall, soluble and microsomal membrane fractions of pea roots. J. Plant Physiol. 161, 1225–1234. 10.1016/j.jplph.2004.02.002 15602814

[B15] Clabeaux B. L. Navarro D. A. Aga D. S. Bisson M. A. (2013). Combined effects of cadmium and zinc on growth, tolerance, and metal accumulation in *Chara australis* and enhanced phytoextraction using EDTA. Ecotoxicol. Environ. Saf. 98, 236–243. 10.1016/j.ecoenv.2013.08.014 24035462

[B16] Cosgrove D. J. Jarvis M. C. (2012). Comparative structure and biomechanics of plant primary and secondary cell walls. Front. Plant Sci. 3:204. 10.3389/fpls.2012.00204 22936943 PMC3424969

[B17] Cosio C. De Santis L. Frey B. Diallo S. Keller C. (2005). Distribution of cadmium in leaves of *Thlaspi caerulescens*. J. Exp. Bot. 56, 765–775. 10.1093/jxb/eri062 15642714

[B18] Crowell E. F. Gonneau M. Stierhof Y. D. Höfte H. Vernhettes S. (2010). Regulated trafficking of cellulose synthases. Curr. Opin. Plant Biol. 13, 700–705. 10.1016/j.pbi.2010.07.005 20822948

[B19] Daković M. Kovacević M. Andjus P. R. Bacić G. (2008). On the mechanism of uranium binding to cell wall of *Chara fragilis*. Eur. Biophys. J. 37, 1111–1117. 10.1007/s00249-008-0282-3 18270692

[B20] Djikanovic D. Kalauzi A. Jeremic M. Xu J. Micic M. Whyte J. D. (2012). Interaction of the CdSe quantum dots with plant cell walls. Colloids Surf. B Biointerfaces 91, 41–47. 10.1016/j.colsurfb.2011.10.032 22104400

[B21] Douchiche O. Driouich A. Morvan C. (2011). Impact of cadmium on early stages of flax fibre differentiation: ultrastructural aspects and pectic features of cell walls. Plant Physiol. Biochem. 49, 592–599. 10.1016/j.plaphy.2011.03.008 21470867

[B22] Douchiche O. Rihouey C. Schaumann A. Driouich A. Morvan C. (2007). Cadmium-induced alterations of the structural features of pectins in flax hypocotyl. Planta 225, 1301–1312. 10.1007/s00425-006-0425-7 17086399

[B23] Douchiche O. Soret-Morvan O. Chaïbi W. Morvan C. Paynel F. (2010). Characteristics of cadmium tolerance in ‘Hermes’ flax seedlings: contribution of cell walls. Chemosphere 81, 1430–1436. 10.1016/j.chemosphere.2010.09.011 20884040

[B24] Du Y. P. Li H. J. Yin K. L. Zhai H. (2012). Cadmium accumulation, subcellular distribution, and chemical forms in *Vitis vinifera* cv. Chardonnay grapevine. Chi. J. Appl. Ecol. 23, 1607–1612. 22937650

[B25] Durand T. C. Sergeant K. Planchon S. Carpin S. Label P. Morabito D. (2010). Acute metal stress in *Populus tremula* × *P. alba* (717-1B4 genotype): leaf and cambial proteome changes induced by cadmium^2+^. Proteomics 10, 349–368. 10.1002/pmic.200900484 20148406

[B26] Engelsdorf T. Hamann T. (2014). An update on receptor-like kinase involvement in the maintenance of plant cell wall integrity. Ann. Bot. 114, 1339–1347. 10.1093/aob/mcu043 24723447 PMC4195549

[B27] Evlard A. Sergeant K. Printz B. Guignard C. Renaut J. Campanella B. (2014a). A multiple-level study of metal tolerance in *Salix fragilis* and *Salix aurita* clones. J. Proteom. 101, 113–129. 10.1016/j.jprot.2014.02.007 24530377

[B28] Evlard A. Sergeant K. Ferrandis S. Printz B. Renaut J. Guignard C. (2014b). Physiological and proteomic responses of different willow clones (*Salix fragilis* × *alba*) exposed to dredged sediment contaminated by heavy metals. Int. J. Phytorem. 16, 1148–1169. 10.1080/15226514.2013.821448 24933908

[B29] Fan J. L. Wei X. Z. Wan L. C. Zhang L. Y. Zhao X. Q. Liu W. Z. (2011). Disarrangement of actin filaments and Ca^2+^ gradient by CdCl_2_ alters cell wall construction in *Arabidopsis thaliana* root hairs by inhibiting vesicular trafficking. J. Plant Physiol. 168, 1157–1167. 10.1016/j.jplph.2011.01.031 21497412

[B30] Fernandes J. C. García-Angulo P. Goulao L. F. Acebes J. L. Amâncio S. (2013). Mineral stress affects the cell wall composition of grapevine (*Vitis vinifera* L.) callus. Plant Sci. 205–206, 111–120. 10.1016/j.plantsci.2013.01.013 23498868

[B31] Fernández R. Fernández-Fuego D. Bertrand A. González A. (2014). Strategies for Cd accumulation in *Dittrichia viscosa* (L.) Greuter: role of the cell wall, non-protein thiols and organic acids. Plant Physiol. Biochem. 78, 63–70. 10.1016/j.plaphy.2014.02.021 24636908

[B32] Ferrari S. Savatin D. V. Sicilia F. Gramegna G. Cervone F. Lorenzo G. D. (2013). Oligogalacturonides: plant damage-associated molecular patterns and regulators of growth and development. Front. Plant Sci. 4:49. 10.3389/fpls.2013.00049 23493833 PMC3595604

[B33] Fry S. C. Nesselrode B. H. W. A. Miller J. G. Mewburn B. R. (2008). Mixed linkage (1–3,1–4)- β-D-glucan is a major hemicellulose of *Equisetum* (horsetail) cell walls. New Phytol. 179, 104–115. 10.1111/j.1469-8137.2008.02435.x 18393951

[B34] Gao J. Sun L. Yang X. Liu J. X. (2013). Transcriptomic analysis of cadmium stress response in the heavy metal hyperaccumulator *Sedum alfredii* Hance. PLoS ONE 8:e64643. 10.1371/journal.pone.0064643 23755133 PMC3670878

[B35] Ge W. Jiao Y. Q. Sun B. L. Qin R. Jiang W. S. Liu D. H. (2012). Cadmium-mediated oxidative stress and ultrastructural changes in root cells of poplar cultivars. S. Afr. J. Bot. 83, 98–108 10.1016/j.sajb.2012.07.026

[B36] Gomes P. I. Asaeda T. (2013). Phytoremediation of heavy metals by calcifying macro-algae (*Nitella pseudoflabellata*): implications of redox insensitive end products. Chemosphere 92, 1328–1334. 10.1016/j.chemosphere.2013.05.043 23773443

[B37] Gu C. S. Liu L. Q. Zhao Y. H. Deng Y. M. Zhu X. D. Huang S. Z. (2014). Overexpression of Iris lactea var. chinensis metallothionein llMT2a enhances cadmium tolerance in *Arabidopsis thaliana*. Ectoxicol. Environ. Safe. 105, 22–28. 10.1016/j.ecoenv.2014.04.002 24780229

[B38] Guerriero G. Sergeant K. Hausman J. F. (2013). Integrated -omics: a powerful approach to understanding the heterogeneous lignification of fibre crops. Int. J. Mol. Sci. 14, 10958–10978. 10.3390/ijms140610958 23708098 PMC3709712

[B39] Guerriero G. Hausman J. F. Cai G. (2014a). No stress! Relax! Mechanisms governing growth and shape in plant cells. Int. J. Mol. Sci. 15, 5094–5114. 10.3390/ijms15035094 24663059 PMC3975442

[B40] Guerriero G. Sergeant K. Hausman J. F. (2014b). Wood biosynthesis and typologies: a molecular rhapsody. Tree Physiol. 34, 839–355. 10.1093/treephys/tpu031 24876292

[B41] Hamann T. Denness L. (2011). Cell wall integrity maintenance in plants: lessons to be learned from yeast? Plant Sign. Behav. 6, 1706–1709 10.4161/psb.6.11.17782 PMC3329341 22067998

[B42] Han Y. L. Yuan H. Y. Huang S. Z. Guo Z. Xia B. Gu J. (2007). Cadmium tolerance and accumulation by two species of Iris. Ecotoxicology 16, 557–563. 10.1007/s10646-007-0162-0 17701346

[B43] Han Y. Sa G. Sun J. Shen Z. Zhao R. Ding M. (2014). Overexpression of *Populus euphratica* xyloglucan endotransglucosylase/hydrolase gene confers enhanced cadmium tolerance by the restriction of root cadmium uptake in transgenic tobacco. Environ. Exp. Bot. 100, 74–83 10.1016/j.envexpbot.2013.12.021

[B44] Harada E. Kim J. A. Meyer A. J. Hell R. Clemens S. Choi Y. E. (2010). Expression profiling of tobacco leaf trichomes identifies genes for biotic and abiotic stresses. Plant Cell Physiol. 51, 1627–1637. 10.1093/pcp/pcq118 20693332

[B45] He J. L. Ma C. F. Ma Y. L. Li H. Kang J. Q. Liu T. X. (2013). Cadmium tolerance in six poplar species. Environ. Sci. Poll. Res. 20, 163–174. 10.1007/s11356-012-1008-8 22669564

[B46] Hradilova J. Rehulka P. Rehulkova H. Vrbova M. Griga M. Brzobohaty B. (2010). Comparative analysis of proteomic changes in contrasting flax cultivars upon cadmium exposure. Electrophoresis 31, 421–431. 10.1002/elps.200900477 20084635

[B47] Humphrey T. V. Bonetta D. T. Goring D. R. (2007). Sentinels at the wall: cell wall receptors and sensors. New Phytol. 176, 7–21. 10.1111/j.1469-8137.2007.02192.x 17803638

[B48] Kieffer P. Dommes J. Hoffmann L. Hausman J. F. Renaut J. (2008). Quantitative changes in protein expression of cadmium-exposed poplar plants. Proteomics 8, 2514–2530. 10.1002/pmic.200701110 18563750

[B49] Kleczkowski L. A. Kunz S. Wilczynska M. (2010). Mechanisms of UDP-glucose synthesis in plants. Crit. Rev. Plant Sci. 29, 191–203 10.1080/07352689.2010.483578

[B50] Konlechner C. Türktas M. Langer I. Vaculík M. Wenzel W. W. Puschenreiter M. (2013). Expression of zinc and cadmium responsive genes in leaves of willow (*Salix caprea* L.) genotypes with different accumulation characteristics. Environ. Pollut. 178, 121–127. 10.1016/j.envpol.2013.02.033 23562959 PMC3675671

[B51] Konno H. Nakato T. Nakashima S. Katoh K. (2005). *Lygodium japonicum* fern accumulates copper in the cell wall pectin. J. Exp. Bot. 56, 1923–1931. 10.1093/jxb/eri187 15928016

[B52] Krzesłowska M. (2011). The cell wall in plant cell response to trace metals: polysaccharide remodeling and its role in defense strategy. Acta Physiol. Plant. 33, 35–51 10.1007/s11738-010-0581-z

[B53] Küpper H. Lombi E. Zhao F. J. McGrath S. P. (2000). Cellular compartmentation of cadmium and zinc in relation to other elements in the hyperaccumulator Arabidopsis halleri. Planta 212, 75–84. 10.1007/s004250000366 11219586

[B54] Kuramata M. Masuya S. Takahashi Y. Kitagawa E. Inoue C. Ishikawa S. (2009). Novel cysteine-rich peptides from *Digitaria ciliaris* and *Oryza sativa* enhance tolerance to cadmium by limiting its cellular accumulation. Plant Cell Physiol. 50, 106–117 10.1093/pcp/pcn175 19017626

[B55] Lagriffoul A. Mocquot B. Mench M. Vangronsveld J. (1998). Cadmium toxicity effects on growth, mineral and chlorophyll contents, and activities of stress related enzymes in young maize plants (*Zea mays* L.). Plant Soil 200, 241–250 10.1023/A:1004346905592

[B56] Lang I. Wernitznig S. (2011). Sequestration at the cell wall and plasma membrane facilitates zinc tolerance in the moss *Pohlia drummondii*. Environ. Exp. Bot. 74, 186–193 10.1016/j.envexpbot.2011.05.018

[B57] Liu D. Jiang W. Gao X. (2003). Effects of cadmium on root growth, cell division and nucleoli in root tip cells of garlic. Biol. Plantar. 47, 79–83 10.1023/A:1027384932338

[B58] Liu D. Kottke I. (2004). Subcellular localization of cadmium in the root cells of *Allium cepa* by electron energy loss spectroscopy and cytochemistry. J. Biosci. 29, 329–335. 10.1007/BF02702615 15381854

[B59] Liu J. Ma J. He C. Li X. Zhang W. Xu F. (2013). Inhibition of cadmium ion uptake in rice (*Oryza sativa*) cells by a wall-bound form of silicon. New Phytol. 200, 691–699. 10.1111/nph.12494 24102436

[B60] Loeffler S. Hochberger A. Grilli E. Gekeler W. Winnacker E. L. Zenk M. H. (1989). Termination of the phytochelatin synthase reactions through sequestration of heavy metals by reaction products. FEBS Lett. 258, 42–46 10.1016/0014-5793(89)81611-4

[B61] Lozano-Rodríguez E. Hernández L. E. Bonay P. Carpena-Ruiz R. O. (1997). Distribution of cadmium in shoot and root tissues of maize and pea plants: physiological disturbances. J. Exp. Bot. 48, 123–128 10.1093/jxb/48.1.123

[B62] Lukacová Z. Švubová R. Kohanová J. Lux A. (2013). Silicon mitigates the Cd toxicity in maize in relation to cadmium translocation, cell distribution, antioxidant enzymes stimulation and enhanced endodermal apoplasmic barrier development. Plant Growth Regul. 70, 89–103 10.1007/s10725-012-9781-4

[B63] Lux A. (2010). Does diversity in root structure affect the diversity in cadmium uptake by plants? Opinion paper. Agrochimica 54, 342–352.

[B64] Lux A. Vaculik M. Martinka M. Liskova D. Kulkarni M. G. Stirk W. A. (2011). Cadmium induces hypodermal periderm formation in the roots of the monocotyledonous medicinal plant *Merwilla plumbea*. Ann. Bot. 107, 285–292. 10.1093/aob/mcq240 21118841 PMC3025738

[B65] Malovikova, A. and Kohn R. (1982). Binding of cadmium cations to pectins. Collec. Czech. Chem. Commun. 47, 702–708.

[B66] Mata Y. N. Blázquez M. L. Ballester A. González F. Muñoz J. A. (2009). Sugar-beet pulp pectin gels as biosorbent for heavy metals: preparation and determination of biosorption and desorption characteristics. Chem. Eng. J. 150, 289–301 10.1016/j.cej.2009.01.001

[B67] Mata Y. N. Blázquez M. L. Ballester A. González F. Munoz J. A. (2010). Studies on sorption, desorption, regeneration and reuse of sugar-beet pectin gels for heavy metal removal. J. Haz. Mat. 178, 243–248. 10.1016/j.jhazmat.2010.01.069 20122797

[B68] Mellerowicz E. J. Gorshkova T. A. (2012). Tensional stress generation in gelatinous fibres: a review and possible mechanism based on cell-wall structure and composition. J. Exp. Bot. 63, 551–565. 10.1093/jxb/err339 22090441

[B69] Mikkelsen M. D. Harholt J. Ulvskov P. Johansen I. E. Fangel J. U. Doblin M. S. (2014). Evidence for land plant cell wall biosynthetic mechanisms in charophyte green algae. Ann. Bot. 114, 1217–1236. 10.1093/aob/mcu171 25204387 PMC4195564

[B70] Mittler R. (2006). Abiotic stress, the field environment and stress combination. Trends Plant Sci. 11, 15–19. 10.1016/j.tplants.2005.11.002 16359910

[B71] Moral R. Gomez I. Pedreno J. N. Mataix J. (1994). Effects of cadmium on nutrient distribution, yield, and growth of tomato grown in soilless culture. J. Plant Nutr. 17, 953–962 10.1080/01904169409364780

[B72] Moussa H. R. El-Gamal S. M. (2010). Role of salicylic acid in regulation of cadmium toxicity in wheat (*Triticum aestivum* L.). J. Plant Nutr. 33, 1460–1471 10.1080/01904167.2010.489984

[B73] Nedelkoska T. V. Doran P. M. (2000). Hyperaccumulation of cadmium by hairy roots of *Thlaspi caerulescens*. Biotech. Bioeng. 67, 607–615 10.1002/(SICI)1097-0290(20000305)67:5<607::AID-BIT11>3.0.CO;2-3 10649235

[B74] Pan X. Liu F. C. Chai M. W. Liu L. M. Morita S. Shi F. C. (2012). Accumulation, translocation, and subcellular distribution of cadmium in *Spartina alterniflora*. Chi. J. Ecol. 31, 526–531.

[B75] Paynel F. Schaumann A. Arkoun M. Douchiche O. Morvan C. (2009). Temporal regulation of cell-wall pectin methylesterase and peroxidase isoforms in cadmium-treated flax hypocotyl. Ann. Bot. 104, 1363–1372. 10.1093/aob/mcp254 19815572 PMC2778398

[B76] Piršelová B. Kuna R. Libantová J. Moravciková J. Matušíkova I. (2011). Biochemical and physiological comparison of heavy metal-triggered defense responses in the monocot maize and dicot soybean roots. Mol. Biol. Rep. 38, 3437–3446. 10.1007/s11033-010-0453-z 21104138

[B77] Piršelová B. Mistríková V. Libantová J. Moravcíková J. Matušíková I. (2012). Study on metal-triggered callose deposition in roots of maize and soybean. Biologia 67, 698–705 10.2478/s11756-012-0051-8

[B78] Podazza C. Rosa M. González J. A. Hilal M. Prado F. E. (2006). Cadmium induces changes in sucrose partitioning, invertase activities, and membrane functionality in roots of rangpur lime (*Citrus limonia* L. *Osbeck*). Plant Biol. 8, 706–714. 10.1055/s-2006-924171 16883481

[B79] Popper Z. A. Fry S. C. (2003). Primary cell wall composition of bryophytes and charophytes. Ann. Bot. 91, 1–12. 10.1093/aob/mcg013 12495914 PMC4240358

[B80] Printz B. Sergeant K. Lutts S. Guignard C. Renaut J. Hausman J. F. (2013a). From tolerance to acute metabolic deregulation: contribution of proteomics to dig into the molecular response of alder species under a polymetallic exposure. J. Proteome Res. 12, 5160–5179. 10.1021/pr400590d 24015726

[B81] Printz B. Sergeant K. Guignard C. Renaut J. Hausman J. F. (2013b). Physiological and proteome study of sunflowers exposed to a polymetallic constraint. Proteomics 13, 1993–2015. 10.1002/pmic.201200400 23595958

[B82] Przedpelska-Wasowicz E. M. Wierzbicka M. (2011). Gating of aquaporins by heavy metals in *Allium cepa* L. epidermal cells. Protoplasma 248, 663–671. 10.1007/s00709-010-0222-9 20960016 PMC3206186

[B83] Qadir S. Jamshieed S. Rasool S. Ashraf M. Akram N. A. Ahmad P. (2014). “Modulation of plant growth and metabolism in cadmium-enriched environments,” in Reviews of Environmental Contamination and Toxicology, ed. Whitacre D. M. (Cham: Springer International Publishing), 51–88 10.1007/978-3-319-03777-6_4 24515810

[B84] Quiroga M. de Forchetti S. M. Taleisnik E. Tigier H. A. (2001). Tomato root peroxidase isoenzymes: kinetic studies of the coniferyl alcohol peroxidase activity, immunological properties and role in response to salt stress. J. Plant Physiol. 158, 1007–1013 10.1078/0176-1617-00304

[B85] Rascio N. Navari-Izzo F. (2011). Heavy metal hyperaccumulating plants: how and why do they do it? And what makes them so interesting? Plant Sci. 180, 169–181. 10.1016/j.plantsci.2010.08.016 21421358

[B86] Redjala T. Sterckeman T. Morel J. L. (2009). Cadmium uptake by roots: contribution of apoplast and of high- and low-affinity membrane transport systems. Environ. Exp. Bot. 67, 235–242 10.1016/j.envexpbot.2009.05.012

[B87] Rodríguez-Serrano M. Romero-Puertas M. C. Pazmiño D. M. Testillano P. S. Risueño M. C. del Río L. A. (2009). Cellular response of pea plants to cadmium toxicity: crosstalk between reactive oxygen species, nitric oxide and calcium. Plant Physiol. 150, 229–243. 10.1104/pp.108.131524 19279198 PMC2675729

[B88] Sandalio L. M. Dalurzo H. C. Gomez M. Romero-Puertas M. C. Del Rio L. A. (2001). Cadmium-induced changes in the growth and oxidative metabolism of pea plants. J. Exp. Bot. 52, 2115–2126. 10.1093/jexbot/52.364.2115 11604450

[B89] Sanità Di Toppi L. Gabbrielli R. (1999). Response to cadmium in higher plants. Environ. Exp. Bot. 41, 105–130 10.1016/S0098-8472(98)00058-6

[B90] Sanità Di Toppi L. Marabottini R. Vattuone Z. Musetti R. Favali M. A. Sorgonà A. (2005). Cell wall immobilisation and antioxidant status of *Xanthoria parietina* thalli exposed to cadmium. Funct. Plant Biol. 32, 611–618 10.1071/FP04237 32689160

[B91] Sarkar P. Bosneaga E. Auer M. (2009). Plant cell walls throughout evolution: towards a molecular understanding of their design principles. J. Exp. Bot. 60, 3615–3635. 10.1093/jxb/erp245 19687127

[B92] Sawidis T. (2008). Effect of cadmium on pollen germination and tube growth in *Lilium longiflorum* and *Nicotiana tabacum*. Protoplasma 233, 95–106. 10.1007/s00709-008-0306-y 18709476

[B93] Sawidis T. Reiss H. D. (1995). Effects of heavy metals on pollen tube growth and ultrastructure. Protoplasma 185, 113–122 10.1007/BF01272851

[B94] Schiewer S. Patil S. B. (2008). Pectin-rich fruit wastes as biosorbents for heavy metal removal: equilibrium and kinetics. Bioresour. Technol. 99, 1896–1903. 10.1016/j.biortech.2007.03.060 17540559

[B95] Schiewer S. Iqbal M. (2010). The role of pectin in Cd binding by orange peel biosorbents: a comparison of peels, depectinated peels and pectic acid. J. Hazard. Mater. 177, 899–907. 10.1016/j.jhazmat.2010.01.001 20122803

[B96] Schützendübel A. Schwarz P. Teichmann T. Gross K. Langenfeld-Heyser R. Godbold D. L. (2001). Cadmium-induced changes in antioxidative systems, hydrogen peroxide content, and differentiation in scots pine roots. Plant Physiol. 127, 887–898. 10.1104/pp.010318 11706171 PMC129260

[B97] Sergeant K. Kieffer P. Dommes J. Hausmann J. F. Renaut J. (2014). Proteomic changes in leaves of poplar exposed to both cadmium and low-temperature. Environ. Exp. Bot. 106, 112–123 10.1016/j.envexpbot.2014.01.007

[B98] Sergio E. Cobianchi R. C. Sorbo S. Conte B. Basile A. (2007). Ultrastructural alterations and HSP 70 induction in *Elodea canadensis* Michx. exposed to heavy metals. Caryologia 60, 115–120 10.1080/00087114.2007.10589557

[B99] Shah K. Singh P. Nahakpam S. (2013). Effect of cadmium uptake and heat stress on root ultrastructure, membrane damage and antioxidative response in rice seedlings. J. Plant Biochem. Biotech. 22, 103–112 10.1007/s13562-012-0116-3

[B100] Siebers N. Siangliw M. Tongcumpou C. (2013). Cadmium uptake and subcellular distribution in rice plants as affected by phosphorus: soil and hydroponic experiments. J. Soil Sci. Plant Nutr. 13, 833–844 10.4067/S0718-95162013005000066

[B101] Siemianowski O. Barabasz A. Kendziorek M. Ruszczynska A. Bulska E. Williams L. E. (2014). HMA4 expression in tobacco reduces Cd accumulation due to the induction of the apoplastic barrier. J. Exp. Bot. 65, 1125–1139. 10.1093/jxb/ert471 24420575 PMC3935570

[B102] Song W. Y. Choi K. S. De Alexis A. Martinoia E. Lee Y. (2011). *Brassica juncea* plant cadmium resistance 1 protein (BjPCR1) facilitates the radial transport of calcium in the root. Proc. Natl. Acad. Sci. U.S.A. 108, 19808–19813. 10.1073/pnas.1104905108 22089235 PMC3241789

[B103] Song X. Q. Liu L. F. Jiang Y. J. Zhang B. C. Gao Y. P. Liu X. L. (2013). Disruption of secondary wall cellulose biosynthesis alters cadmium translocation and tolerance in rice plants. Mol. Plant 6, 768–780. 10.1093/mp/sst025 23376772

[B104] Sørensen I. Pettolino F. A. Bacic A. Ralph J. Lu F. O’Neill M. A. (2011). The charophycean green algae provide insights into the early origins of plant cell walls. Plant J. 68, 201–211. 10.1111/j.1365-313X.2011.04686.x 21707800

[B105] Soudek P. Katrusáková A. Sedlácek L. Petrová S. Kocí V. Marsík P. (2010). Effect of heavy metals on inhibition of root elongation in 23 cultivars of flax (*Linum usitatissimum* L.). Arch. Environ. Contam. Toxicol. 59, 194–203. 10.1007/s00244-010-9480-y 20174789

[B106] Souza V. L. De Almeida A. A. F. Lima S. G. C. De M. C. Da C. S. Mangabeira P. A. O. (2011). Morphophysiological responses and programmed cell death induced by cadmium in *Genipa americana* L. (Rubiaceae). Biometals 24, 59–71. 10.1007/s10534-010-9374-5 20838856

[B107] Sun J. Cui J. Luo C. Gao L. Chen Y. Shen Z. (2013). Contribution of cell walls, nonprotein thiols, and organic acids to cadmium resistance in two cabbage varieties. Arch. Environ. Contam. Toxicol. 64, 243–252. 10.1007/s00244-012-9824-x 23111495

[B108] Suzuki N. (2005). Alleviation by calcium of cadmium-induced root growth inhibition in *Arabidopsis* seedlings. Plant Biotech. 22, 19–25 10.5511/plantbiotechnology.22.19

[B109] Uegsegger A. Schmuts D. Brunold C. (1990). Regulation of glutathione synthesis by cadmium in *Pisum sativum* L. Plant Physiol. 93, 1579–1584 10.1104/pp.93.4.1579 16667659 PMC1062714

[B110] Ueki S. Citovsky V. (2005). Identification of an interactor of cadmium ion-induced glycine-rich protein involved in regulation of callose levels in plant vasculature. Proc. Natl. Acad. Sci. U.S.A. 102, 12089–12094. 10.1073/pnas.0505927102 16103368 PMC1189354

[B111] Uraguchi S. Kiyono M. Sakamoto T. Watanabe I. Kuno K. (2009). Contributions of apoplasmic cadmium accumulation, antioxidative enzymes and induction of phytochelatins in cadmium tolerance of the cadmium-accumulating cultivar of black oat (*Avena strigosa* Schreb.). Planta 230, 267–276. 10.1007/s00425-009-0939-x 19437035

[B112] Vallarino J. G. Osorio S. (2012). Signaling role of oligogalacturonides derived during cell wall degradation. Plant Sign. Behav. 7, 1447–1449. 10.4161/psb.21779 22918501 PMC3548869

[B113] van Bakel H. Stout J. M. Cote A. G. Tallon C. M. Sharpe A. G. Hughes T. R. (2011). The draft genome and transcriptome of *Cannabis sativa*. Genome Biol. 12, R102. 10.1186/gb-2011-12-10-r102 22014239 PMC3359589

[B114] Vázquez S. Goldsbrough P. Carpena R. O. (2006). Assessing the relative contributions of phytochelatins and the cell wall to cadmium resistance in white lupin. Physiol. Plant. 128, 487–495 10.1111/j.1399-3054.2006.00764.x

[B115] Verma K. Shekhawat G. S. Sharma A. Mehta S. K. Sharma V. (2008). Cadmium induced oxidative stress and changes in soluble and ionically bound cell wall peroxidase activities in roots of seedling and 3-4 leaf stage plants of *Brassica juncea* (L.) czern. Plant Cell Rep. 27, 1261–1269. 10.1007/s00299-008-0552-7 18449543

[B116] Wan L. Zhang H. (2012). Cadmium toxicity effects on cytoskeleton, vesicular trafficking and cell wall construction. Plant Sign. Behav. 7, 345–348. 10.4161/psb.18992 22499203 PMC3443916

[B117] Wan Ngah W. S. Hanafiah M. A. K. M. (2008). Removal of heavy metal ions from wastewater by chemically modified plant wastes as adsorbents: a review. Bioresour. Technol. 99, 3935–3948. 10.1016/j.biortech.2007.06.011 17681755

[B118] Wang X. Gao Y. Feng Y. Li X. Wei Q. Sheng X. (2014). Cadmium stress disrupts the endomembrane organelles and endocytosis during *Picea wilsonii* pollen germination and tube growth. PLoS ONE 9:e94721. 10.1371/journal.pone.0094721 24722362 PMC3983259

[B119] Wang Y. Huang J. Gao Y. (2012). Arbuscular mycorrhizal colonization alters subcellular distribution and chemical forms of cadmium in *Medicago sativa* L. and resists cadmium toxicity. PLoS ONE 7:e48669. 10.1371/journal.pone.0048669 23139811 PMC3490862

[B120] Webster E. A. Gadd G. M. (1996). Cadmium replaces calcium in the cell wall of *Ulva lactuca*. Biometals 9, 241–244 10.1007/BF00817922

[B121] Weng B. Xie X. Weiss D. J. Liu J. Lu H. Yan C. (2012). *Kandelia obovata* (S., L.) Yong tolerance mechanisms to cadmium: subcellular distribution, chemical forms and thiol pools. Mar. Pollut. Bull. 64, 2453–2460. 10.1016/j.marpolbul.2012.07.047 22910331

[B122] Wierzbicka M. H. Przedpelska E. Ruzik R. Ouerdane L. Polec-Pawlak K. Jarosz M. (2007). Comparison of the toxicity and distribution of cadmium and lead in plant cells. Protoplasma 231, 99–111. 10.1007/s00709-006-0227-6 17370112

[B123] Wolf S. Hematy K. Hofte H. (2012). Growth control and cell wall signaling in plants. Ann. Rev. Plant Biol. 63, 381–407. 10.1146/annurev-arplant-042811-105449 22224451

[B124] Wolff G. Pereira G. C. Castro E. M. Louzada J. Coelho F. F. (2012). The use of *Salvinia auriculata* as a bioindicator in aquatic ecosystems: biomass and structure dependent on the cadmium concentration. Braz. J. Biol. 72, 71–77. 10.1590/S1519-69842012000100009 22437387

[B125] Wong C. K. E. Cobbett C. S. (2009). HMA P-type ATPases are the major mechanism for root-to-shoot Cd translocation in *Arabidopsis thaliana*. New Phytol. 181, 71–78. 10.1111/j.1469-8137.2008.02638.x 19076718

[B126] Wu G. Kang H. Zhang X. Shao H. Chu L. Ruan C. (2010). A critical review on the bio-removal of hazardous heavy metals from contaminated soils: issues, progress, eco-environmental concerns and opportunities. J. Hazard. Mater. 174, 1–8. 10.1016/j.jhazmat.2009.09.113 19864055

[B127] Xin J. Huang B. Yang J. Yang Z. Yuan J. Mu Y. (2013). Role of roots in cadmium accumulation of two water spinach cultivars: reciprocal grafting and histochemical experiments. Plant Soil 366, 425–432 10.1007/s11104-012-1439-5

[B128] Xing J. P. Jiang R. F. Ueno D. Ma J. F. Schat H. McGrath S. P. (2008). Variation in root-to-shoot translocation of cadmium and zinc among different accessions of the hyperaccumulators *Thlaspi caerulescens* and *Thlaspi praecox*. New Phytol. 178, 315–325. 10.1111/j.1469-8137.2008.02376.x 18266619

[B129] Xiong J. An L. Lu H. Zhu C. (2009). Exogenous nitric oxide enhances cadmium tolerance of rice by increasing pectin and hemicellulose contents in root cell wall. Planta 230, 755–765. 10.1007/s00425-009-0984-5 19626338

[B130] Xiong J. Fu G. Tao L. Zhu C. (2010). Roles of nitric oxide in alleviating heavy metal toxicity in plants. Arch. Biochem. Biophys. 497, 13–20. 10.1016/j.abb.2010.02.014 20193657

[B131] Xu J. Jia R. Shi G. X. Tian X. L. Yang H. Y. Xu X. Y. (2012a). Subcellular distribution and phytotoxicity of cadmium in *Alternanthera philoxeroides* leaves. Chi. J. Appl. Ecol. 23, 1070–1076. 22803476

[B132] Xu J. Sun J. Du L. Liu X. (2012b). Comparative transcriptome analysis of cadmium responses in *Solanum nigrum* and *Solanum torvum*. New Phytol. 196, 110–124. 10.1111/j.1469-8137.2012.04235.x 22809404

[B133] Xu W. Shi W. Yan F. Zhang B. Liang J. (2011). Mechanisms of cadmium detoxification in cattail (*Typha angustifolia* L.). Aquat. Bot. 94, 37–43 10.1016/j.aquabot.2010.11.002

[B134] Yamaguchi N. Mori S. Baba K. Kaburagi-Yada S. Arao T. Kitajima N. (2011). Cadmium distribution in the root tissues of solanaceous plants with contrasting root-to-shoot Cd translocation efficiencies. Environ. Exp. Bot. 71, 198–206 10.1016/j.envexpbot.2010.12.002

[B135] Yang J. Pan X. (2013). Root exudates from sunflower (*Helianthus annuus* L.) show a strong adsorption ability toward Cd(II). J. Plant Interact. 8, 263–270 10.1080/17429145.2012.737030

[B136] Zhang J. Shu W. S. (2006). Mechanisms of heavy metal cadmium tolerance in plants. J. Plant Physiol. Mol. Biol. 32, 1–8. 16477124

[B137] Zhang J. Wu H. Wang X. Huang W. (2010a). Subcellular distribution and chemical forms of cadmium in the cells of strawberry (*Fragaria × ananassa* Duch.). J. Hortic. Sci. Biotechnol. 85, 563–569.

[B138] Zhang Z. C. Chen B. X. Qiu B. S. (2010b). Phytochelatin synthesis plays a similar role in shoots of the cadmium hyperaccumulator *Sedum alfredii* as in non-resistant plants. Plant Cell Environ 33, 1248–1255. 10.1111/j.1365-3040.2010.02144.x 20233337

[B139] Zhang Q. Yan C. Liu J. Lu H. Duan H. Du J. (2014a). Silicon alleviation of cadmium toxicity in mangrove (*Avicennia marina*) in relation to cadmium compartmentation. J. Plant Growth Regul. 33, 233–242 10.1007/s00344-013-9366-0

[B140] Zhang S. J. Li T. X. Huang H. G. Zhang X. Z. Yu H. Y. Zheng Z. C. (2014b). Phytoremediation of cadmium using plant species of *Athyrium wardii* (Hook.). Int. J. Environ. Sci. Tech. 11, 757–764 10.1007/s13762-013-0384-z

[B141] Zhang W. Lin K. Zhou J. Zhang W. Liu L. Zhang Q. (2014c). Cadmium accumulation, sub-cellular distribution and chemical forms in rice seedling in the presence of sulfur. Environ. Toxicol. Pharmacol. 37, 348–353. 10.1016/j.etap.2013.12.006 24388908

[B142] Zhimin Y. Shaojian Z. Aitang H. U. (1999). Subcellular accumulation of cadmium in corn and wheat plants at different levels of phosphorus. Pedosphere 9, 169–176.

[B143] Zhou B. Yao W. Wang S. Wang X. Jiang T. (2014). The metallothionein gene, TaMT3, from Tamarix androssowii confers Cd^2+^ tolerance in tobacco. Int. J. Mol. Sci. 15, 10398–10409. 10.3390/ijms150610398 24918294 PMC4100158

[B144] Zhu X. F. Lei G. J. Jiang T. Liu Y. Li G. X. Zheng S. J. (2012). Cell wall polysaccharides are involved in P-deficiency-induced Cd exclusion in *Arabidopsis thaliana*. Planta 236, 989–997. 10.1007/s00425-012-1652-8 22526505

